# Outbreak of an Emerging Recombinant of Myxoma Virus ha‐MYXV in European Brown Hares (*Lepus europaeus*) in Austria

**DOI:** 10.1155/tbed/7125710

**Published:** 2026-09-28

**Authors:** Angelika Auer, Sarah Kugler, Melina Papadopoulos, Katharina Dimmel, Janina Steiner, Joachim Spergser, Julia Reichl, Anna-Margarita Schötta, Hans-Peter Fuehrer, Georg Gerhard Duscher, Till Rümenapf, Anna Kübber-Heiss

**Affiliations:** ^1^ Department of Biological Sciences and Pathobiology, Infectiology and Virology, University of Veterinary Medicine, Veterinaerplatz 1, Vienna, Austria, vetmeduni.ac.at; ^2^ Department of Interdisciplinary Life Sciences, Research Institute of Wildlife Ecology, University of Veterinary Medicine, Savoyenstraße 1, Vienna, Austria, vetmeduni.ac.at; ^3^ Department of Biological Sciences and Pathobiology, Microbiology, University of Veterinary Medicine, Veterinaerplatz 1, Vienna, Austria, vetmeduni.ac.at; ^4^ Institute for Medical Microbiology and Hygiene, AGES–Austrian Agency for Health and Food Safety, Vienna, Austria, ages.at; ^5^ Department of Biological Sciences and Pathobiology, Parasitology, University of Veterinary Medicine, Veterinaerplatz 1, Vienna, Austria, vetmeduni.ac.at; ^6^ Institute for Veterinary Disease Control Moedling, AGES–Austrian Agency for Health and Food Safety, Moedling, Austria, ages.at

**Keywords:** ha-MYXV, lagomorphs, myxomatosis, poxvirus, vectors

## Abstract

Myxomatosis, caused by the myxoma virus (MYXV), is a severe disease primarily affecting European rabbits (*Oryctolagus cuniculus*), with very high case fatality rates. Historically, MYXV was introduced to Europe and Australia in the 1950s to control rabbit populations, becoming endemic and undergoing genetic adaptations. In 2018, a novel recombinant variant, hare‐adapted MYXV (ha‐MYXV), emerged in Spain, causing significant declines in Iberian hare (*Lepus granatensis*) populations. This study reports the first outbreak of ha‐MYXV in Austria, severely affecting European brown hares (*Lepus europaeus*) and wild rabbits during 2025. Between March and November 2025, 272 hare and 12 rabbit carcasses were submitted for investigation. Pathological examinations revealed characteristic swelling and inflammation around the eyes, nose, and genital tract, accompanied by histological epithelial changes consistent with a poxvirus infection in 235 European brown hares and 10 wild rabbits from 89 municipalities in Austria. PCR analysis and cytopathic effects (CPEs) observed in rabbit kidney 13 (RK13) cell culture confirmed the presence of MYXV. Sequencing further identified a typical 2.8 kb M009L gene insertion, definitively linking the outbreak to the highly pathogenic ha‐MYXV variant. The outbreak spread across Vienna, Lower Austria, and Burgenland, with hunters reporting hundreds of additional unsubmitted cases. The emergence of ha‐MYXV in Austria highlights the virus’s geographic expansion, possibly facilitated by human‐mediated transport of infected animals or vectors. The outbreak also underscores the potential indirect One Health risks posed by coinfections with zoonotic bacteria in affected hares and emphasizes the need for enhanced surveillance, genomic analysis, more targeted screening of potential vectors, and cross‐border collaboration to monitor the evolution and spread of ha‐MYXV, mitigate its impact on wildlife populations, and address associated public health concerns.

## 1. Introduction

Myxomatosis is known as a severe and aggressive disease that, in its classical form, leads to large skin nodules and swellings on the head and in the genital area of European rabbits (*Oryctolagus cuniculus*). The case fatality rates vary in a strain‐dependent manner but can reach up to 100% [1]. Transmission can occur through direct contact, but it is more likely that the virus is spread by blood‐sucking insects such as fleas, mosquitoes, and ticks. As members of the poxvirus family (genus *Leporipoxvirus*), myxoma viruses (MYXVs) are large, enveloped DNA viruses [2]. The linear, double‐stranded 161.8 kb genome comprises 171 open reading frames coding for 165–171 genes [3]. Isolated from its American well‐adapted reservoir hosts (genus *Sylvilagus*), the virus exhibited exceptionally high virulence in European rabbits [1] and was released in Australia and Europe to control invasive and local rabbit populations in the 1950s. Since then, the virus has become endemic, causing recurring outbreaks among both wild and domestic rabbit populations in Australia and in many European countries. Meanwhile, it has undergone numerous genetic adaptations to its “new” host over the past decades [4, 5]. Apart from isolated cases [6, 7], until a few years ago, MYXV occurred exclusively in rabbits. In 2018 a novel, hare‐adapted MYXV (ha‐MYXV) emerged in Spain and resulted in a remarkable decline in the Iberian hare (*Lepus granatensis*) population across the whole Iberian Peninsula [8–12]. Genetic analyses discovered 110 mutations as well as one 2.8 kb insertion within the M009L gene if compared to the MYXV reference strain Lausanne [8].

Introduction of the pathogen to the European brown hare population was confirmed in northern Spain in 2020 [13]. A further outbreak in 2023 and 2024 in the area around the German–Dutch border [14] showed the continued spread of the pathogen in Europe.

The European brown hare (*Lepus europaeus*) plays a crucial ecological role, serving as key prey for various carnivorous predators while also significantly influencing the environment through its herbivorous activities. Furthermore, the European brown hare is an important game species in Austria. The largest populations of hares are in Lower Austria (where the recent outbreak commenced), followed by Upper Austria and Burgenland. The Austrian hunting harvest of hares in 2024 was 104,792 animals plus additional 25,183 losses on the street or through diseases [15]. Various viral and bacterial pathogens, such as lagoviruses (European brown hare syndrome virus [EBHSV] and rabbit hemorrhagic disease virus 2 [RHDV2]), as well as zoonotic bacteria such as *Francisella tularensis*, *Brucella suis*, and *Yersinia* spp. can pose a serious threat to European brown hare populations [16, 17]. However, the MYXV was never considered to be one of these pathogens in Central Europe so far.

In 2025, an outbreak of myxomatosis in European brown hares and rabbits caused by ha‐MYXV was observed and investigated in Austria. Following this outbreak, the virus also spread to Austria’s neighboring countries, the Czech Republic, Slovakia, and Hungary [18, 19].

## 2. Materials and Methods

### 2.1. Outbreak Description, Necropsy, and Sampling Procedure

Starting on March 26, 2025, the Pathology Lab of the Research Institute of Wildlife Ecology (University of Veterinary Medicine) received a growing number of hare carcasses. These animals, primarily originating from Vienna, Lower Austria, and Burgenland, were collected by hunters. The submission of animals was recommended but not mandatory. Moribund hares showed noticeable clinical signs, including swelling and inflammation around the eyes, nasal discharge, diarrhea, disorientation, and a lack of flight reflex. However, most of the animals were found dead and showed macroscopic changes typical of myxomatosis.

Carcasses of dead lagomorphs submitted for postmortem examination from March 26 until the end of 2025 were included in this study. Date and provenance, along with age (categorized as adult [>1 year] or juvenile [<1 year if the “Stroh sign,” a cartilaginous thickening of the distal ulnar epiphysis, was still present]), sex, and weight were recorded. Histological samples were prepared according to standard protocols. Skin samples of macroscopically altered regions such as eyelids, nose, and prepuce or vagina were collected and sent to the Unit for Infectiology and Virology (Department of Biological Sciences and Pathobiology, University of Veterinary Medicine) for molecular detection of MYXV. In case of macroscopic alterations of lungs, liver, and/or spleen, samples were additionally sent to respective laboratories for bacteriological investigations.

At the beginning of the outbreak, samples of all hares and rabbits suspected of myxomatosis were submitted for virological confirmation. As the outbreak progressed and due to the high number of cases, hunters were instructed to send only carcasses from areas not yet confirmed as positive and to dispose of all other carcasses at designated collection points. Virological investigations were restricted to confirmation of virus spread to new regions and to questionable necropsy cases.

### 2.2. DNA Extraction, PCR Amplification, and Sequencing

At the Unit for Infectiology and Virology, tissue samples from altered skin areas were analyzed for the presence of MYXV‐specific nucleic acids. For nucleic acid extraction, ~100 mg of tissue was lysed in 1 mL of sterile PBS using four sterile 2.8 mm stainless steel beads and the TissueLyser II (Qiagen, Hilden, Germany). The lysate was then centrifuged at 16,000 × g for 1 min, and 300 µL of the supernatant was processed using the TANBead Maelstrom 4800 system and the TANBead Nucleic Acid Extraction Kit (Taiwan Advanced Nanotech, Taiwan), following the manufacturer’s instructions.

Sample extracts were screened by using a primer pair (A1F 5′‐TCA TTC TAA AAC AGG ACG GGA‐3′ and A1R 5′‐TGT TTC GGT TAA ACG ACG CT‐3′) published in 2010 [20] amplifying a 658 bp long fragment of the M137R gene of MYXV. Conventional PCR was carried out employing the Fast Cycling PCR Kit (Qiagen, Hilden, Germany). Cycling conditions were 95°C for 5 min, followed by 45 cycles of 96°C for 5 s, 60°C for 5 s, 68°C for 21 s, and a final extension at 72°C for 1 min. The PCR product was analyzed by using a QIAxcel Advanced capillary electrophoresis system (Qiagen, Hilden, Germany).

To distinguish between classic MYXV and the ha‐MYXV variant, from selected cases, a PCR targeting the M009L gene was performed [8]. The presence or absence of the 2845 bp insert, characteristic of the ha‐MYXV variant, was determined based on the length of the PCR product. The primers M009L‐F (5′‐CGC AGG TCC ACG TAT AAA CC‐3′) and M009L‐R (5′‐CGA ACG TAT CAT TAG ACA ATG‐3′) were used in PCR reactions prepared with Phanta Max Master Mix (Vazyme, Nanjing, PRC) at concentrations specified by the manufacturer but in a reduced total reaction volume of 25 µL, which included 2 µL of template DNA. The cycling conditions were performed according to the manufacturer’s protocol, with an annealing temperature of 58°C and an extension time of 3 min and 30 s per cycle.

Subsequently, 6 µL of the PCR products was analyzed on a 0.8% agarose gel stained with Midori Green (Nippon Genetics Europe GmbH, Germany). The remaining PCR products were purified using PCR Kleen Spin Columns (Bio‐Rad, Austria) and submitted for sequencing (Eurofins Genomics AT GmbH). The obtained nucleotide sequences were compared to reference sequences (AF170726, MYXV strain Lausanne, and MK340973, MYXV strain Tol08‐18, also referred to as ha‐MYXV) from the GenBank database (NCBI) using Clone Manager 9.

For faster analysis of samples, published qPCR primer and probe sets were adopted during the outbreak [21].

To confirm the viability and infectivity of the pathogen, the tissue lysate of one sample was sterile filtered (0.45 µm), and 10 mL was inoculated onto a subconfluent monolayer (6.9 cm^2^) of rabbit kidney 13 (RK13) cells. The cells were then examined under a microscope at 10× magnification on days 4, 5, and 7 for the presence of cytopathic effect (CPE) characteristic of MYXVs.

The ha‐MYXV cell culture isolate from sample 358_25 was whole‐genome sequenced using the following protocol: Cell culture supernatant was concentrated by ultracentrifugation at 30,000 × g for 1 h, and 100 µL, including the pellet, was extracted using the DNeasy Blood & Tissue Kit (Qiagen, Hilden, Germany). The concentration of the eluate was determined by fluorometry using Qubit 4 fluorometer (Thermo Scientific). For nanopore sequencing (Oxford Nanopore Technologies, UK), libraries were prepared using the Ligation Sequencing Kit SQK‐LSK114 and barcoded using the Native Barcoding Kit SQK‐NBD114. Sequencing was performed on a MinION Mk1D device equipped with a FLO‐MIN114 flow cell. Base calling was carried out using MinKNOW version 25.05.12 with the integrated Dorado base‐calling software. Resulting FASTQ files were processed in Geneious Prime 2025.02 (Biomatters Ltd., New Zealand), and reads shorter than 1000 bases were removed.

The virus isolate was additionally sequenced on an Illumina MiniSeq platform (2 × 150 bp). Sequencing libraries were prepared using the Illumina DNA Prep (M) Tagmentation Kit. Raw sequencing data were processed in Geneious Prime 2025.02 and quality‐filtered using the BBDuk plugin. Adapter sequences were removed, and reads were retained only if they had a Phred quality score > 30 and a minimum length of 100 bp after trimming.

Illumina short reads and nanopore long reads were subsequently subjected to hybrid genome assembly using Unicycler version 0.5.1 with default parameters [22], resulting in a complete viral genome sequence that was deposited in GenBank under Accession Number PZ495198.

For phylogenomic analysis, complete genomes of classical and ha‐MYXV isolates were retrieved from GenBank and aligned together with strain SK358_25 using MAFFT (version 7.490), as implemented in Geneious Prime 2025.02. Pairwise evolutionary distances were estimated under the HKY85 nucleotide substitution model, and a Neighbor‐Joining (NJ) tree was reconstructed from the resulting distance matrix using Geneious Tree Builder. Node support was evaluated using 1000 bootstrap replicates.

### 2.3. Vector Monitoring

One thousand seven hundred sixty‐one mosquitoes and 1432 ticks from the ha‐MYXV outbreak areas, which were collected and extracted for the Austrian vector monitoring program of the Austrian Agency for Health and Food Safety in 2025, were analyzed using qPCR (primer and probe set targeting the M000.5 gene) published by Dos Santos et al. [21]. The 183 tick pools contained a total of 1432 individual tick nucleic acid extracts (pool size 6–8 samples) from a citizen science collection comprising of the following tick species: *Ixodes ricinus* (*n* = 1195), *Ixodes hexagonus* (*n* = 15), *Ixodes acuminatus* (*n* = 3), *Ixodes frontalis* (*n* = 1), *Ixodes vespertilionis* (*n* = 1), *Ixodes* spp. (*n* = 105), *Dermacentor marginatus* (*n* = 1), *Dermacentor reticulatus* (*n* = 78), *Dermacentor* spp. (*n* = 1), and *Haemaphysalis concinna* (*n* = 32). Within the pools, a minimum of one tick was derived from the myxomatosis‐affected regions.

Moreover, a total of 1761 adult mosquitoes (280 pools to 1–50 mosquitoes), including the genera *Culex* (*n* = 1564), *Aedes* (*n* = 108), *Anopheles* (*n* = 43), *Coquillettidia* (*n* = 30), *Culiseta* (*n* = 2), *Orthopodomyia* (*n* = 1), and other Culicinae (*n* = 13), were collected using BG‐Sentinel‐traps (with CO_2_ and lure as bait) as part of the mosquito monitoring programs within the provinces of Vienna and Lower Austria. The identification of the species was determined by morphological analysis.

## 3. Results

### 3.1. Temporal Dynamics and Geographical Spread of the Outbreak

Between March 26 and November 27, 2025, 272 deceased hares and 12 wild rabbits were sent in from over 89 different political municipalities. The time course of the first occurrence of the disease in different regions is shown in Figure 1 and Table 1. Of 272 hares examined, 233 showed macroscopic signs typical of myxomatosis. Two animals could not be assessed pathologically due to postmortem changes but tested positive for ha‐MYXV in PCR, and 37 had no lesions. Eleven of those macroscopically negative animals were tested, and none of them yielded positive results. Ten out of 12 wild rabbits had typical lesions.

**Figure 1:**
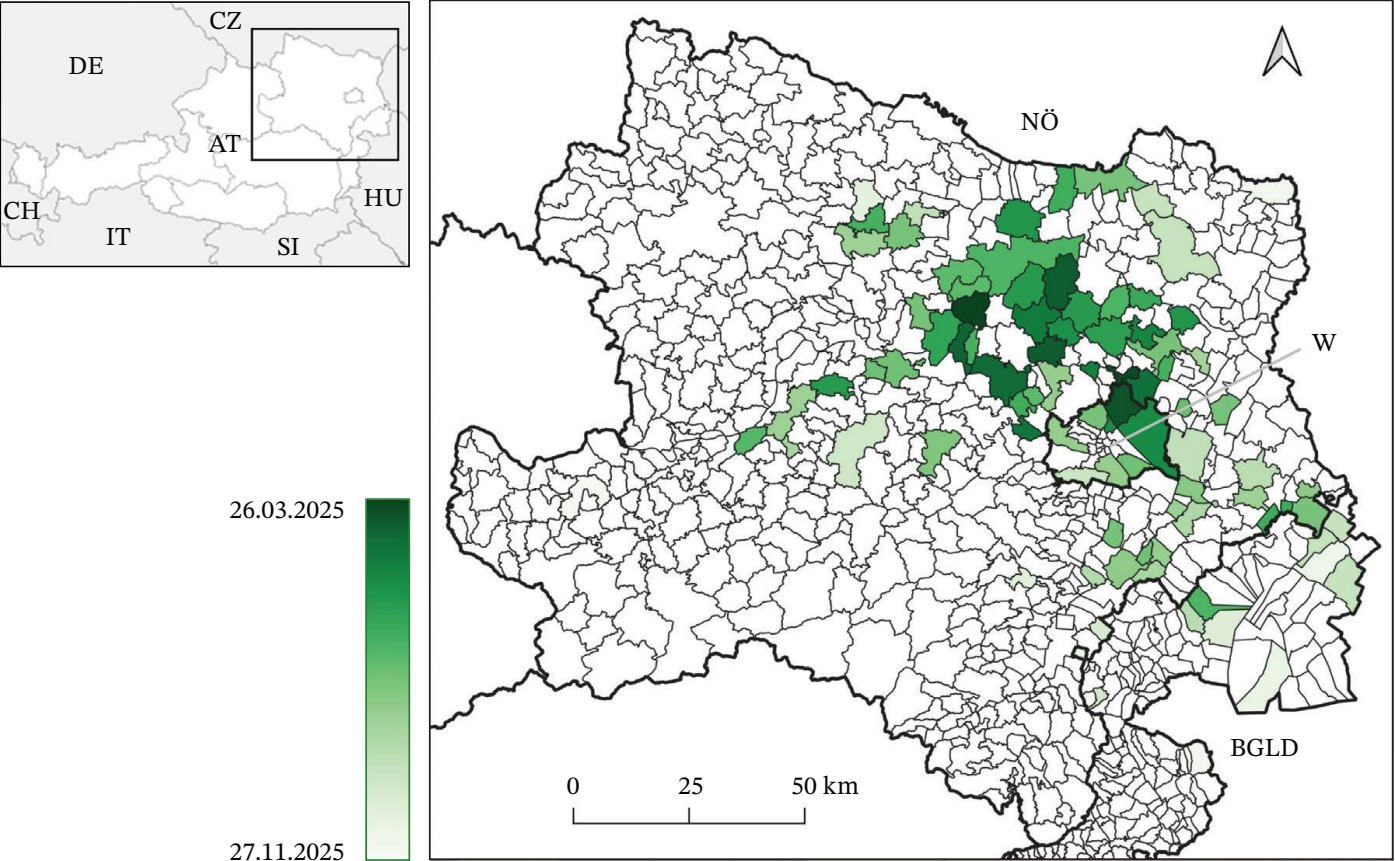
Distribution of PCR‐positive lagomorphs in municipalities of Lower Austria (NÖ), Vienna (W), and Burgenland (BGLD) from 26.03.2025 (dark green) to 27.11.2025 (light green). Map created in QGIS 3.40.10 using country outlines from data.gv.at.

**Table 1:** Temporal dynamics: number of infected carcasses and spread to new municipalities per month in 2025.

Month	European brown hares	Wild rabbits	New municipalities
March	1	0	1
April	2	0	2
May	9	0	3
June	10	1	1
July	95	9	17
August	57	0	33
September	29	0	20
October	15	0	5
November	17	0	7
December	0	0	0

As the submission was voluntary and depended on hunter initiative, the map does not reflect the true incidence of the disease. White areas between affected municipalities may have been impacted as well, but no animals were submitted. The outbreak began in spring with 12 cases across six municipalities and rose sharply over the summer, peaking in July. Transmission continued through August, with 33 newly affected municipalities that month alone, and then declined during the colder months, with no cases reported in December (Table 1).

### 3.2. Macroscopic and Microscopic Findings

All infected animals were severely emaciated. The distribution of sex and age is shown in Table 2. Examined lagomorphs were mostly adult individuals, and an equal sex ratio was observed in hares. The main macroscopic lesion observed in nearly all examined lagomorphs was eyelid swelling, in various degrees and nodular to diffuse, with mucopurulent conjunctivitis and corneal ulcer and edema in severe cases. The presence of edema of the nasal, oral, and genital orifices with mucopurulent discharge and nodular skin proliferations varied between cases (Figure 2). Other macroscopic findings were tracheal and pulmonary hyperemia or hemorrhage, purulent pneumonia, and splenomegaly. Macroscopic lesions did not differ between hares and rabbits.

**Figure 2:**
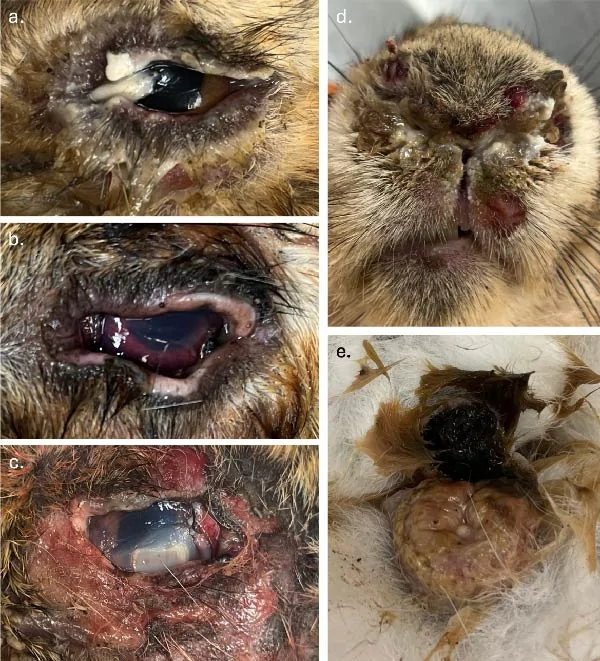
Macroscopic findings (*Lepus europaeus*). (a) Moderate nodular eyelid swelling and mucopurulent conjunctivitis. (b) Severe diffuse eyelid swelling and ocular hemorrhage. (c) Severe eyelid swelling, periocular ulcerations, and corneal ulcer. (d) Nasal and oral edema, nodular skin proliferations with ulceration, and mucopurulent discharge. (e) Severe edema of the male genital mucous membranes.

**Table 2:** Distribution of sex and age of infected carcasses.

	Sex		Age	
	Female	Male	Juvenile	Adult
*Lepus europaeus* (*n* = 235)	121	114	19	216
	51%	49%	8%	92%
*Oryctolagus cuniculus* (*n* = 10)	3	7	1	9
	30%	70%	10%	90%

Microscopic examination showed similar lesions in the skin, including adnexa, and in mucous membranes of the eyelids and the oronasal and genital region. Epithelial changes included epidermal hyperplasia, hyperkeratosis, hydropic degeneration of keratinocytes, and eosinophilic intracytoplasmic inclusion bodies. The dermis contained myxoma cells, fibroblasts, heterophils, and mononuclear inflammatory cells embedded in myxoid stroma and edema. Ulceration was most commonly found at mucocutaneous junctions with suppurative to necrotizing inflammation and frequent bacterial colonies (Figure 3). Wild rabbits showed more frequent intracytoplasmic inclusion bodies than European brown hares.

**Figure 3:**
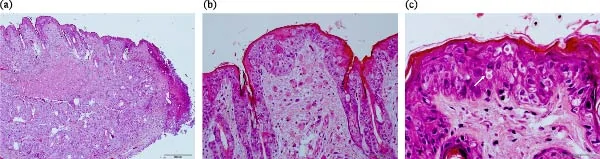
Microscopic findings, eyelid. (a) Hyperplastic epidermis and ulceration of the mucocutaneous junction, European brown hare, H&E, 40×. (b) Hyperplastic epidermis with hyperkeratosis, hydropic degeneration, and myxoma cells and heterophils in the dermis, European brown hare, H&E, 200×. (c) Hydropic degeneration and intracytoplasmic inclusion bodies (arrow) in keratinocytes, wild rabbit, H&E, 600×.

### 3.3. Virological Findings

To date, MYXV‐specific DNA has been detected in 144 out of 156 European brown hare samples and in seven out of eight wild rabbit samples, which were sent for virological investigation. With the exception of two cadavers that could not be assessed pathologically, all PCR‐positive animals exhibited macroscopic lesions typical of myxomatosis. Twelve samples came from lagomorphs without macroscopic lesions (11 hares and one rabbit), and they were all MYXV‐PCR negative. Only one suspected case, where only the inner organs were available for testing, could not be confirmed by molecular analysis. Table 3 provides an overview of the molecular biology studies conducted and their results.

**Table 3:** Overview of the molecular biology studies and results of the myxoma virus (MYXV) PCR.

Clinical signs typical of myxomatosis		MYXV PCR (*n* = 156)			No MYXV PCR (*n* = 116)
		Tested	Positive	Negative	
*Lepus europaeus* (*n* = 272)					
Yes	233	143	142	1	90
No	37	11	0	11	26
Not assessable	2	2	2	0	0
Total	272	156	144	12	116
*Oryctolagus cuniculus* (*n* = 12)					
Yes	10	7	7	0	3
No	2	1	0	1	1
Total	12	8	7	1	4

Additionally, in 81 MYXV PCR‐positive lagomorphs—at least one per municipality—ha‐MYXV–specific qPCR was performed, and the cycle threshold (Ct) values ranged from 16.61 (minimum) to 39.67 (maximum), with a mean of 20.95 and a median of 19.00. Four MYXV‐positive rabbits were tested for the ha‐MYXV insertion, and it was detected in all cases. Three DNA products obtained from the strain‐differentiating PCR [8] were sequenced, and the resulting fragments, each 4603 bp in length, showed 100% identity when compared to MK340973 (Figure 4). The viability and infectivity of the virus were confirmed by virus isolation using RK13) cells; a typical CPE could be observed as shown in Figure 5.

**Figure 4:**
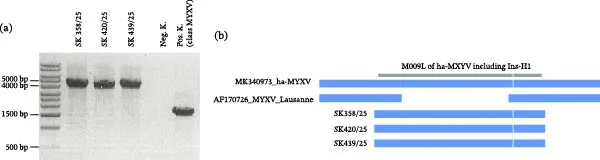
(a) Agarose gel electrophoresis analyses of three PCR products from strain‐differentiating PCR. The length of amplified fragments is 1758 bp for the classical myxoma virus strain and 4603 bp for ha‐MYXV. (b) Location map of alignment showing MK340973 (ha‐MYXV; genome position 10,000–17,520) and AF170726 (MYXV strain Lausanne, genome position 10,023–14,699). Sample sequences clearly indicating the presence of the 2.8 kb insert (Ins‐H1) in the M009L gene region.

**Figure 5:**
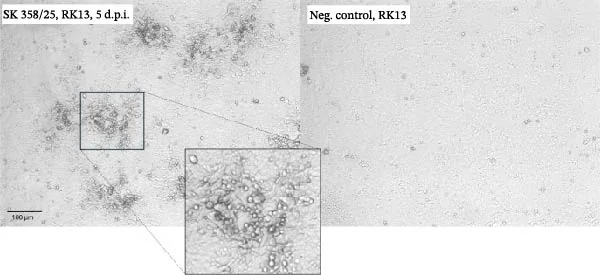
Images of ha‐MYXV–infected RK13 cells 5 days after infection (left) and negative control (right).

Phylogenetic analysis of complete genome sequences revealed a clear separation between classical MYXV strains and ha‐MYXV strains (Figure 6). The Austrian strain SK358_25 clustered within the ha‐MYXV lineage and was clearly distinct from the classical MYXV group. Within the ha‐MYXV clade, SK358_25 grouped most closely with a ha‐MYXV strain detected in the Czech Republic in 2025, indicating a high degree of genomic similarity between the two viruses. The clustering pattern was supported by bootstrap analysis.

**Figure 6:**
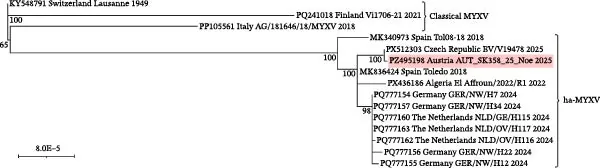
Neighbor‐Joining (NJ) phylogenetic tree based on complete genome sequences of representative classical myxoma virus (MYXV) and hare‐adapted myxoma virus (ha‐MYXV) isolates. The classical MYXV strain Lausanne (KY548791) was used as the outgroup. Bootstrap support values, calculated from 1000 replicates, are shown at the corresponding branch nodes as percentages.

No ha‐MYXV–specific DNA could be detected in any of the vector pools—neither in mosquitoes nor in ticks.

## 4. Discussion

In spring 2025, the emergence of ha‐MYXV in Austria was confirmed, reflecting similar outbreaks previously observed in hare populations in Spain [11], Portugal [10], and the German–Dutch border area [14]. The presence of the ha‐MYXV variant was also recently documented in the Czech Republic in August 2025, Slovakia in October 2025 [18], and Hungary in October 2025 [19]. There is also a report of ha‐MYXV in farmed rabbits from Algeria in 2022 [23].

Starting with the first hare cases in the surrounding of Vienna, the virus spread continuously to several districts in Lower Austria and Burgenland. Since myxomatosis is not a notifiable disease, dead lagomorphs (primarily European brown hares) were voluntarily sent to the Research Institute of Wildlife Ecology for investigation. Whether carcasses were submitted and how many largely depended on the personal interest of the responsible hunters. The fact that some municipalities on the distribution map (Figure 1) remained white, despite being surrounded by affected zones, suggests that the collection of dead animals for further examination was not very stringent. Although carcasses of affected hares are missing from several regions, hunters reported the disposal of hundreds of dead hares that were visibly infected with the MYXV.

How exactly hare‐associated MYXV (ha‐MYXV) spreads among European brown hares (*L. europaeus*) remains unclear and should be the subject of further investigations. As no cases of myxomatosis in European brown hares have been previously reported in Austria, it was straightforward to assume a recent introduction of the 2018 Spanish MYXV variant. The outbreak observed here started in March 2025, and similar to the hypothesis proposed by Fischer et al. [14] for the German–Dutch epidemic, it might be related to the import of infected lagomorphs or vectors by human transportation. It can be surmised that other vectors, such as certain species of fleas, could act as competent vectors due to the rapid spread of this pathogen within the population. Although most mosquitoes and ticks have a preference for feeding on certain host species, they are not typically as closely associated with the host species as fleas are. However, sources such as ticks carried by migratory birds cannot be ruled out either. Ectoparasites were rarely encountered in the animals examined in this study and were therefore not analyzed further. Garcia‐Pereira et al. [24] detected MYXV‐specific DNA in 50.7% of tick pools collected in Spain from 27 wild rabbits and three hares, some of which were confirmed to be infected with MYXV. Beyond this, there are few data on the vector competence of ticks in MYXV transmission. The 183 tick pools collected in the areas affected by the ha‐MYXV outbreak documented here yielded uniformly negative qPCR results. Because most of these samples were submitted by citizen scientists, it is quite possible that the ticks had not been in contact with rabbits or hares suffering from myxomatosis prior to testing. In this study, 280 mosquito pools were screened for MYXV‐specific nucleic acids, and all tested negative. Frequent mechanical transmission of classical MYXV by mosquitoes has been described by Fenner et al. [25] and Day et al. [26] and appears to play an important role in rabbit transmission in Australia. The rabbit flea (*Spilopsyllus cuniculi*), who effectively transmits classical MYXV in the British rabbit population, can hardly be found in hare species and was not detected on the animals included in this study. The activity of different potential vectors also depends on climatic conditions; Austrian weather data are available on the websites of Geosphere Austria [27]. Interestingly, the cases in the lagomorphs accumulate in the months July, August, and September, which aligns with the period of highest mosquito´s activity over the course of a year, whereas during the hotter months, tick activity usually decreases. For fleas circulating, for example, on rabbits in dens, climate might have less impact on flea numbers, although an accumulation over the course of the year can be expected. These observations hint at mosquitoes or fleas as presumed vectors, but this needs further evaluation and investigations on vector competences, which can give an integral picture on actual vector capacity of the arthropods in focus.

Individual hunters reported the presence of dead European brown hares days to weeks before the first animals were submitted, further complicating the temporal determination of virus introduction. The first cases of ha‐MYXV in regions close to the affected Austrian regions were detected in August 2025 in the Czech Republic and in October 2025 in Slovakia as well as in West‐Hungary [18, 19]. Given the timeline and the high genetic similarity between the Austrian and Czech ha‐MYXV sequences, the Central European outbreak is consistent with an origin in eastern Austria. Still, the possibility of unreported cases in other parts of Europe over the past year cannot be ruled out.

Most wildlife fatalities caused by ha‐MYXV predominantly involve European brown hares, whereas wild rabbits are affected to a much lesser extent, as supported by data from Spain and Germany [11, 14]. However, a report from Portugal [28] as well as an infection study from Spain [29] states a high virulence of the ha‐MYXV variant in rabbits. This fits with a mass die‐off of local Viennese rabbit colonies, which was observed in spring and summer 2025. The population consisted partly of wild rabbits and partly of abandoned domestic rabbits as well as hybrids. Numerous reports of ill and dead animals showing macroscopic changes typical of myxomatosis were documented by local animal welfare associations, and many private photographs were uploaded to social media groups. Unfortunately, only a small number of animals were investigated virologically; however, in all those animals, the ha‐MYXV variant was detected, while lagoviruses (RHDV1 and RHDV2) were ruled out (data not shown). According to the company responsible for disposal of dead animals within Vienna (ebswien kläranlage & tierservice Ges.m.b.H.), 743 carcasses of deceased lagomorphs were disposed of between April and October 2025, peaking in July. This was 3.5 times as many as in the same period of the previous year (personal communication). In recreational areas, particularly those in peri‐urban regions, the presence of animal carcasses not only raised concerns within the hunting community but also drew significant attention from hikers and walkers. This is also accompanied by an increased risk that careless individuals, children, or free‐roaming dogs could come into direct contact with the dead animals, potentially leading to infection with zoonotic bacteria (e.g., *B. suis* and *F. tularensis*), which are also endemic in Austrian hares. The presence of such pathogens was not systematically investigated within this study, but suspected organs were sent for bacteriological investigation and occasionally yielded positive results. Thus, hares that have died from myxomatosis do not represent a direct one‐health issue but to a certain extent an indirect one.

Vaccination of pet, breeding, and meat rabbits against myxomatosis is recommended by the Standing Committee on Vaccination in Veterinary Medicine (StIKo Vet) of the Friedrich‐Loeffler‐Institut (FLI) [30]. From the current perspective, large‐scale vaccination of wild animals is neither feasible nor advisable. While commercial vaccines do protect rabbits against the ha‐MYXV variant, a study by Dos Santos et al. [29] found that only one of the three vaccines tested was able to induce protection in Iberian hares when administered at higher doses. No comparable data are available for the European brown hare; furthermore, a vaccine suitable for such a mass immunization campaign targeting wild lagomorphs would need to be orally administered and specifically ingested by the target species. Safety is also a concern here: Introducing live, replicating poxviruses while various wild‐type leporipoxviruses are circulating could have unforeseeable consequences with regard to the emergence of new recombinant species. Ultimately, a cost–benefit analysis would not support such a project. Myxomatosis is not a zoonotic disease nor is the European hare an endangered species.

Pathology findings were consistent with those of recent outbreaks in hares. Eyelid swelling and nodular skin proliferations predominately on mucocutaneous junctions were reported as the most prominent lesions in European brown as well as Iberian hares, and there is no obvious difference in disease manifestation between European brown and Iberian hare species [14, 18, 19, 31]. Classic MYXV has never been isolated from a European brown hare in Austria before, and there was also no coinfection with ha‐MYXV during the study period. A single case report from the United Kingdom reports classic MYXV in a European brown hare. The classic disease mainly manifested in the haired skin around the ears and the eyes, compared to a focus on mucocutaneous junctions in ha‐MYXV–infected hares. Notably, all animals examined in this study were severely emaciated or cachectic compared to only 21.2% in Germany and the Netherlands [14]. Moreover, Iberian hares were reported to be in good body condition in 92.8% of cases [31]. The clarity of the macroscopic lesions, particularly those observed on the eyelids, facilitates straightforward and dependable diagnosis. In all but one instance, samples from animals exhibiting macroscopic signs of infection consistently tested positive by PCR. However, a retrospective examination of the PCR‐negative case revealed that no skin sample had been preserved. Instead, an organ pool was used for PCR analysis, which is considered suboptimal for detecting MYXV DNA.

To date, several methods for detection and differentiation of MYXV have been published. All methods applied in this study proved to be comparably reliable and easy to perform, likely due to the high viral load present in the majority of affected tissues.

Single cases of myxomatosis in European brown hares have been documented occasionally [6], and a case of myxomatosis in an Italian hare (*Lepus corsicanus*) has been published in 2024 in Italy [7]. Sequence analysis of this hare MYXV showed several genetic differences compared to the Lausanne reference strain, but the 2.8 kb M009L gene insert was absent. This insertion was first documented in Spain in 2018 in Iberian hares, signifying the species jump from rabbits to hares. In our study, the detection of the insertion for confirming the ha‐MYXV variant was performed using conventional M009L‐PCR published by Dalton et al. [8] and qPCR methods (m060L) published by Dos Santos et al. [21]. Dalton et al. [8] compared whole‐genome sequence data and provided detailed information about several mutations between the Lausanne reference strain and the ha‐MYXV variant. The species switch from the Iberian hare to the European brown hare was once again accompanied by evolutionary changes in the viral genome, as demonstrated for the German–Dutch outbreak [14]. Phylogenetic analyses of the whole‐genome sequence obtained during this Austrian outbreak indicate ongoing viral evolution and underscore the need for continued surveillance.

In conclusion, the emergence and spread of the ha‐MYXV variant in Austria underscores the need for improved monitoring, genomic analysis, and collaboration to better understand the virus’s transmission and evolution, mitigate its impact on wildlife, and address potential public health concerns by monitoring zoonotic coinfections such as tularemia and brucellosis.

## Funding

Part of this study was cofunded by the EU4Health Work Programme CP‐g‐22‐04.01 (HaDEA) (Grant 101132974) as part of the project OneHealth SURVector (mosquito and tick samples). Views and opinions expressed are, however, those of the authors only and do not necessarily reflect those of the European Union or HaDEA. Neither the European Union nor the granting authority can be held responsible for them.

## Ethics Statement

An ethical statement is not applicable as samples were collected from dead animals or from animals legally hunted by authorized hunters with the correct permits and license and with the permission of landowners.

## Conflicts of Interest

The authors declare no conflicts of interest.

## Data Availability

The genomic information of the ha‐MYXV strain AUT_SK358_25_Noe 2025 was submitted to GenBank under the Accession Number PZ495198.
